# Robotic Technology as a Tool for Precision Psychiatric Medicine: A Brief Review

**DOI:** 10.1192/j.eurpsy.2026.11866

**Published:** 2026-07-31

**Authors:** N. Bouayed Abdelmoula, B. Abdelmoula

**Affiliations:** LR23ES07 Genomics of Signalopathies at the service of Precision Medicine, Medical University of Sfax, Sfax, Tunisia

## Abstract

**Introduction:**

Precision psychiatry look for tailoring mental health interventions according to individual biological and clinical characteristics. Robotic technology has emerged as a promising complement to improve the accuracy of diagnosis and personalized treatments delivery in this area.

**Objectives:**

This mini-review aims to explore and synthesize the current advances in robotic technologies that support the development of precision psychiatry.

**Methods:**

This review summarizes the recent developments in robotic applications relevant to precision psychiatry, with an emphasis on neuromodulation, therapeutic robotics and the integration of artificial intelligence for tailor-made psychiatric care. A literature review was conducted using peer-reviewed sources published over the past five years, focusing on translational research and clinical applicability.

**Results:**

Our review demonstrated the powerful role of robotics, which represents a valuable catalyst in precision psychiatric medicine, facilitating adapted and effective mental health care through improved neuromodulation and therapeutic support. Robotic systems integrated into neuromodulation therapies, such as robot-assisted transcranial magnetic stimulation, improve targeting accuracy and treatment reproducibility by adjusting the positioning of the coil in real time according to neuroimaging and patient-specific movements. Social care robots offer supportive roles in therapy, providing engagement and behavioral intervention for disorders such as autism and dementia. In addition, integration with AI improves individualized data-driven treatment planning. While microscopic robotic devices for direct interventions on the brain remain experimental, current robotic technologies significantly advance the objectives of precision psychiatry.

**Conclusions:**

Robotic tools contribute to the establishment of personalized psychiatric care by improving the specificity of the intervention, consistency and interaction with the patient. The challenges include technical complexity, accessibility and ethical considerations. Ongoing research aims to optimize robotic-human integration in order to maximize clinical benefits.

**Disclosure of Interest:**

None Declared

